# Associations Between Oral Microbiota Pathogens and Elevated Depressive and Anxiety Symptoms in Men

**DOI:** 10.1155/da/9961595

**Published:** 2025-07-21

**Authors:** Fannie Kerff, Julie A. Pasco, Lana J. Williams, Felice N. Jacka, Amy Loughman, Samantha L. Dawson

**Affiliations:** ^1^Institute for Mental and Physical Health and Clinical Translation (IMPACT), School of Medicine and Barwon Health, Deakin University, Geelong, VIC, Australia; ^2^Laboratory of Food Systems Biotechnology, Institute of Food, Nutrition, and Health, ETH Zurich, Zurich, Switzerland; ^3^Department of Medicine, The University of Melbourne – Western Campus, St. Albans, VIC, Australia; ^4^Department of Epidemiology and Preventive Medicine, Monash University, Melbourne, VIC, Australia; ^5^Centre for Adolescent Health, Murdoch Children's Research Institute, Melbourne, VIC, Australia; ^6^College of Public Health, Medical and Veterinary Sciences, James Cook University, Townsville, QLD, Australia; ^7^Melbourne School of Psychological Sciences, The University of Melbourne, Melbourne, VIC, Australia

**Keywords:** anxiety, major depressive disorder, mental disorders, oral microbiota

## Abstract

Systemic inflammation is associated with depression. Certain oral bacterial species contribute to inflammation; however their potential association with mental disorders remains unclear. This study investigated the associations between oral microbiota pathogens and depressive and anxiety symptoms. Data came from 436 men from the Geelong Osteoporosis Study. Oral microbiota was characterized using 16S rRNA sequencing, and an oral pathogen composite was created comprising *Porphyromonas gingivalis*, *Treponema denticola*, *Fusobacterium nucleatum*, and *Prevotella nigrescens* species relative abundances. Binary variables were created representing elevated depressive and anxiety symptoms using the Hospital Anxiety and Depression Scale. Logistic regression was used to investigate associations between oral pathogens and elevated depressive/anxiety symptoms. Models were adjusted for confounders: age, socioeconomic status, diet, smoking, alcohol, exercise, obesity, and hypertension. We report a modest (nonsignificant) association between the pathogen composite and elevated depressive (OR 1.35 [95% CI 0.974, 1.87]) but not anxiety symptoms. Moreover, some of the comprising species were significantly associated with elevated depressive symptoms, including *P. nigrescens* (1.61 [1.21, 2.13]). Our exploratory analyses revealed that several other taxa were significantly associated with depression and anxiety symptoms. The findings suggest that specific oral bacteria may contribute to symptoms of depression, warranting further research through larger and longitudinal investigations.

## 1. Introduction

Major depressive disorder and anxiety disorders are common mental disorders that constitute a significant global burden, representing the 13th and 24th leading causes of global disability-adjusted life years, respectively [1]. Cross-sectional studies associate systemic inflammation with elevated depressive and anxiety symptoms [2]. Additionally, longitudinal studies indicate that higher levels of inflammation (interleukin 6, C-reactive protein) precede the onset of depressive symptoms, depressive disorders, psychotic experiences, and psychotic disorder [3–6]. The gut microbiota has emerged as a potential modulator of brain function and behavior, affecting depressive and anxiety symptoms, interacting via the microbiota–gut–brain axis with stress and inflammatory pathways [7]. The oral microbiota is directly connected to the gut microbiota via the gastrointestinal tract [8] and may have a direct route from the oral cavity to the brain, via the trigeminal nerve [9] and/or the blood-brain barrier [10]. Oral bacteria produce virulence factors—such as lipopolysaccharides and gingipains from *Porphyromonas gingivalis—*that may promote inflammatory and neurotoxic effects [11, 12]. The association between the oral microbiota and depression and anxiety disorders, collectively referred to as common mental disorders, requires further investigation.

A small number of studies have investigated differences in the oral microbiota in those with depressive and/or anxiety symptoms compared to controls [2, 13–16]. These typically have small sample sizes, and not all studies report differences [16]. Healthy, nonsmoking adults with higher distress exhibited greater salivary microbiota diversity compared to those with lower distress [14]. Whereas in a study in adolescents, specific bacterial taxa in the oral microbiota were associated with anxiety and depressive symptoms, but there were no differences in diversity [2]. The family, order, and phylum of the genus *Treponema*, namely *Spirochaetaceae*, *Spirochaetales*, and *Spirochaetes*, were all positively associated with the severity of depressive and anxiety symptoms in adolescents. Compared to controls matched for age, sex, and—where possible—smoking status, young adults with depression had a greater abundance of *Prevotella nigrescens* and the genus *Neisseria*, as well as differences in beta (but not alpha) diversity [15]. Depressive phases of bipolar affective disorders have been associated with elevated levels of *Aggregatibacter actinomycetemcomitans* and *P. gingivalis* [13].

Alzheimer's disease shares some similar inflammatory and neuroimmune pathophysiology with the common mental disorders [10, 17], with particular oral pathogens implicated in both. For example, *P. gingivalis* [11] and their lipopolysaccharides [12] infected the cerebrospinal fluid and the brain of post-mortem Alzheimer's disease patients. A seven-times greater load of *Spirochaetes* was also detected in brains from people with Alzheimer's disease [18], suggesting potential interactions of *P. gingivalis* with pathogenic *Treponema* taxa such as *Treponema denticola* [19, 20]. Moreover, the presence of the *Treponema* genus in Alzheimer's patients' trigeminal nerves suggested one of its potential routes of access to the brain [9]. Furthermore, *Klebsiella pneumoniae* and *Fusobacterium nucleatum* have been implicated in inflammation and may be associated with extraoral inflammatory comorbidities [21].

This study aimed to investigate the association between the oral microbiota, focusing on the candidate species identified above, and elevated depressive and/or anxiety symptoms in participants from an Australian cohort. We hypothesized that these candidate oral species are positively associated with elevated depressive and/or anxiety symptoms. While prior work suggested a role of the oral microbiome in adolescents with depressive symptoms [2] and extended this to young adults with clinical diagnoses [15], our study broadened the scope to middle-aged adults, including both depressive and less-studied anxiety symptoms, and replicated the previously reported associations with candidate oral pathogens.

## 2. Methods

This study was preregistered in May 2023 on the Open Science Framework platform (https://osf.io/wcfkp/). This study was approved by the Human Research Ethics Committee at Barwon Health (ID 00/56, July 25, 2023). Therefore, the work described has been carried out in accordance with the Declaration of Helsinki, and all procedures were performed in compliance with relevant laws and institutional guidelines. Written informed consent was obtained from all participants.

### 2.1. Study Design and Sample

The Geelong Osteoporosis Study (GOS) is an ongoing, observational cohort study of adults randomly selected from the electoral roll. Inclusion criterion was a listing as a resident of the Barwon Statistical Division in south-eastern Australia; residency in the region for less than 6 months and inability to provide informed consent necessitated exclusion [22]. This study utilizes cross-sectional data from 436 men who provided an oral sample as part of the 15-year male follow-up, conducted from 2016 to 2020. Women were not included in this analysis, as oral samples were not available at the time of writing. Participants were included in the analysis dataset if they had complete data for the exposure (gum swab) and outcome (depressive and anxiety symptoms). Men were excluded if they had a past-year diagnosis of severe disease (including cancer, heart, and brain diseases), due to the potential nontrivial effects of these conditions on the oral microbiota and depressive/anxiety symptoms.

### 2.2. Oral Microbiota Exposure Measures

#### 2.2.1. Collection and Processing

Oral microbiota samples were collected via swabs of the upper and lower gum-line using sterile cotton tips. The samples were stored in a −80°C freezer located at the University Hospital Geelong. We used bacterial 16S ribosomal RNA (16S rRNA) gene-based next-generation sequencing (NGS) to profile the bacterial composition. Library preparation and sequencing was conducted at Charles River Laboratory (Australia), using two sets of 16S primers 27F-336R (V1-V2) and 341F-785R (V3-V4). For sequencing, the NovaSeq SP 500 cycle flow cell (NV058 and NV058RE2) 251 | 10 | 10 | 251 was used.

Oral microbiota data were analyzed using the R package *phyloseq* (v1.44.0). No filtering of low abundant or low prevalent features was performed for the main analysis, given the need to extract the maximum number of operational taxonomic units (OTUs) from candidate pathogens in the samples. After agglomerating the oral taxa at the species level, a centered log ratio (CLR) transformation was performed on all oral taxa as per best practice [23]. The CLR transformation of the data was performed using the CLR transform function of the *Tjazi* package [24], reflecting how OTUs perform relative to the per-sample average [25]. The zero imputation was implemented using the Martın-Fernandez et al. method, meaning replacing all zeros with 65% of the detection limit, threshold minimizing the distortion in the covariance structure [26]. This approach was chosen over alternatives such as Bayesian multiplicative replacement for its transparency, lower variability in imputed values, and suitability for compositional data [27].

#### 2.2.2. Oral Pathogen Composite

To investigate candidate oral species hypothesized to be associated with elevated depressive and/or anxiety symptoms, a multiple-species composite score was computed following a compositional data analysis approach, henceforth referred to as the pathogen composite. The rationale behind employing this method lies in the understanding that microbes operate in functional guilds rather than in isolation [28], and microbiota studies are often underpowered [29]. All components of the composite are interdependent features that cannot be fully understood in isolation [25]. A correlation matrix of all species in the composite was created in order to assess multicollinearity between species and identify species not positively correlated with the composite.

To build this pathogen composite, the CLR transformed abundances of the OTUs corresponding to candidate oral species were retrieved and summed into one value per sample, reflecting its pathogen composite score. The candidate oral pathogens listed in the preregistration of this study included *P. gingivalis*, *F. nucleatum*, *T. denticola*, *A. actinomycetemcomitans*, and *K. pneumoniae*. Since then, evidence of an association between oral *P. nigrescens* and clinical depression has arisen [15], and therefore these were also included in the pathogen composite.

#### 2.2.3. Microbial Diversity

The richness, diversity, and degree of variation of the oral microbiota were quantified from the raw count table using the alpha diversity indices Chao1, Simpson, and Shannon entropy. Following a prevalence filtering set at 1% to remove rare taxa, beta diversity was evaluated using principal component analysis (PCA) based on the Aitchison distance [30] (i.e., Euclidean distance over CLR-transformed values [24]).

### 2.3. Outcome Measures

#### 2.3.1. Depressive and Anxiety Symptoms

Depressive and anxiety symptoms were assessed using the 14-item Hospital Anxiety and Depression Scale (HADS) questionnaire, a self-report measure of symptom frequency over the past week [31]. HADS scores 0–7 represent “normal”, 8–10 “mild”, 11–14 “moderate”, and 15–21 “severe” symptoms [32]. The cutoff score at ≥8 for both HADS-A and HADS-D has been shown to provide a balance between sensitivity and specificity (i.e., sensitivities and specificities for both subscales are ~0.8) and provides a suitable screening threshold [31].

#### 2.3.2. Primary Outcome

We created a binary case group defined as HADS-D scores of ≥8, representing “elevated depressive symptoms”, in line with consensus in previous literature [31]; scores less than 8 represented “minimal depressive symptoms”.

#### 2.3.3. Secondary Outcome

Similarly, HADS-A scores were dichotomized, with HADS-A scores ≥8 representing “elevated anxiety symptoms” [31]; scores under 8 represented “minimal anxiety symptoms”.

### 2.4. Covariates

The associations between the oral microbiota and the presence of elevated depressive/anxiety symptoms were modeled using a causal inference modeling framework for observational data [33]. A directed acyclic graph was used to identify potential confounders for depressive and anxiety symptoms [34] and the oral microbiota: age [35], socioeconomic status (SES) [36], diet [37], smoking [38, 39], current alcohol intake [40], physical activity [41], and the presence of a chronic disorder, with most evidence on obesity [35, 42] and hypertension [38] (included in the pre-registration; Supporting Information Figure S1). SES was determined using the deciles (1–10) of the Index of Relative Socioeconomic Advantage and Disadvantage (IRSAD), a component of the SocioEconomic Indexes for Areas (Australian Bureau of Statistics). To measure the inflammatory potential of participants' diets, the Energy-adjusted Dietary Inflammatory Index (E-DII) was computed [43, 44] based on responses from the Dietary Questionnaire for Epidemiological Studies (DQES) developed by the Cancer Council Victoria [45]. A lifestyle risk score (0–5) was defined using the information from current alcohol intake, smoking, and physical activity [46]. A detailed description of its computation is included in Supporting Information Table S1. Obesity was defined as having a body mass index of ≥30 (World Health Organization). Hypertension was self-reported by participants. Missing covariate data were imputed to the median of the cohort.  Table 1 provides a summary of the covariates included in the study.

### 2.5. Statistical Analysis

Statistical analyses were performed using R (v4.3.0); statistical significance was determined at the 5% level.

#### 2.5.1. Main Analysis

Logistic regressions (R package *stats* (v4.3.0)) were computed to measure the association between the oral pathogens, first together as a composite, then individually, and elevated depressive and anxiety symptoms. Both unadjusted and adjusted models were created, the latter controlling for covariates (age, SES, diet, lifestyle risk (smoking, alcohol intake, and exercise), obesity, and hypertension). The logistic regression models measured how a change of one standard deviation in the oral bacteria exposure (CLR transformed) was related to the odds of having elevated depressive or anxiety symptoms. The composite was normalized before the regression modeling.

#### 2.5.2. Exploratory Analysis

Alpha diversity metrics were calculated using the R package *Tjazi* (0.1.0.0). To investigate beta diversity, an analysis of similarities (ANOSIM) was performed using the R package *vegan* (v 2.6.4).

After applying a prevalence filtering of 10%, differential abundance analyses between all oral microbiota species in the samples and elevated depressive and anxiety symptoms were performed using the *MaAsLin2* package [47], both in unadjusted and adjusted models. *MaAsLin2* parameters included a CLR transformation, to align with the main analyses, and a statistical significance set at a *p*-value < 0.05 and a *q*-value < 0.1, after applying a Benjamini–Hochberg adjustment for multiple testing [48].

## 3. Results

### 3.1. Descriptive Characteristics of the Included Participants

Four hundred and thirty-six participants were included in the study (flow chart in Supporting Information Figure S2; details of the excluded conditions in Supporting Information Table S2). Participants had a median age of 62 years and a median IRSAD decile score of 6, indicating moderate levels of relative advantage and disadvantage (Table 2). The median E-DII was −0.07, indicating a diet that is neither distinctly anti-inflammatory nor proinflammatory, and the average lifestyle risk was 2 out of a possible score of 0–5, representing a relatively low risk sample regarding smoking, alcohol use, and lack of physical activity. Obesity was recorded in 26.2% of participants, and 33.7% had hypertension. Thirty-nine men (8.9%) had elevated depressive symptoms, and 66 (15.1%) had elevated anxiety symptoms (Table 2).

### 3.2. Oral Pathogen Composite

The swab samples included information on 900 unique OTUs. We included in the composite the candidate oral species that were detected in the samples. *K. pneumoniae* species were not included as no OTU assigned to them was detected; similarly, we did not include *A. actinomycetemcomitans* species and *F. nucleatum subsp. fusiforme ATCC 51190* species as these were poorly detected in the samples and not positively correlated with the composite (details in Supporting Information Table S3; correlation matrix in Supporting Information Figure S3). After retrieving the OTUs of the candidate pathogens and computing their correlations, the pathogen composite reflected the loads of *P. gingivalis*, *T. denticola*, *F. nucleatum* (*F. nucleatum subsp. vincentii ATCC 49256*, *F. nucleatum subsp. vincentii 3_1_36A2*, *F. nucleatum subsp. vincentii*, *F. nucleatum subsp. animalis 7_1*, *F. nucleatum subsp. polymorphum*, F. *nucleatum subsp. animalis ATCC 51191* and *F. nucleatum subsp. animalis*) and *P. nigrescens* (*P. nigrescens ATCC 33563*) species. The CLR transformation of the data resulted in a near-normal distribution of the pathogen composite score (Supporting Information Figure S4).

### 3.3. Associations Between the Oral Pathogen Composite and Elevated Depressive and Anxiety Symptoms

**Figure 1:**
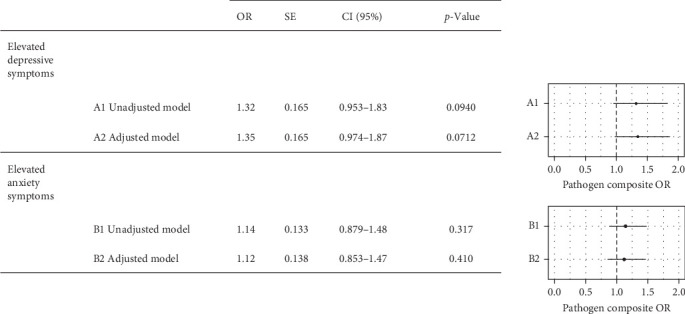
Logistic regressions result for the main analysis: association between the oral pathogen composite and elevated depressive and anxiety symptoms. Modest (nonsignificant) association between the pathogen composite and elevated depressive symptoms (*N* = 39), compared to minimal depressive symptoms (*N* = 397), both in unadjusted (*p*=0.0940) and adjusted (*p*=0.0712) models. No association of the pathogen composite between participants with elevated (*N* = 66) versus minimal (*N* = 370) anxiety symptoms, both in unadjusted and adjusted models (*p*  > 0.05). Adjusted models control for confounders: age, SES, diet, lifestyle risk, obesity, and hypertension. The pathogen composite reflects the combined load of *P. gingivalis*, *T. denticola*, *F. nucleatum subsp. vincentii ATCC 49256*, *F. nucleatum subsp. vincentii 3_1_36A2*, *F. nucleatum subsp. vincentii*, *F. nucleatum subsp. animalis 7_1*, *F. nucleatum subsp. polymorphum*, *F. nucleatum subsp. animalis ATCC 51191, F. nucleatum subsp. animalis*, and *P. nigrescens ATCC 33563*. Error bars represent 95% CI. 95% CI, 95% confidence interval; OR, odds ratio estimate; SE, standard error; SES, socioconomic status.

There was a modest, nonsignificant association between the pathogen composite score and the presence of elevated depressive symptoms, both in unadjusted and adjusted models (OR [odds ratio] 1.35 (95% CI [95% confidence interval] 0.974, 1.87), Figure 1). There were no associations observed between the oral pathogen composite score and elevated anxiety symptoms.

### 3.4. Associations Between Oral Species Within the Pathogen Composite and Elevated Depressive and Anxiety Symptoms

**Figure 2:**
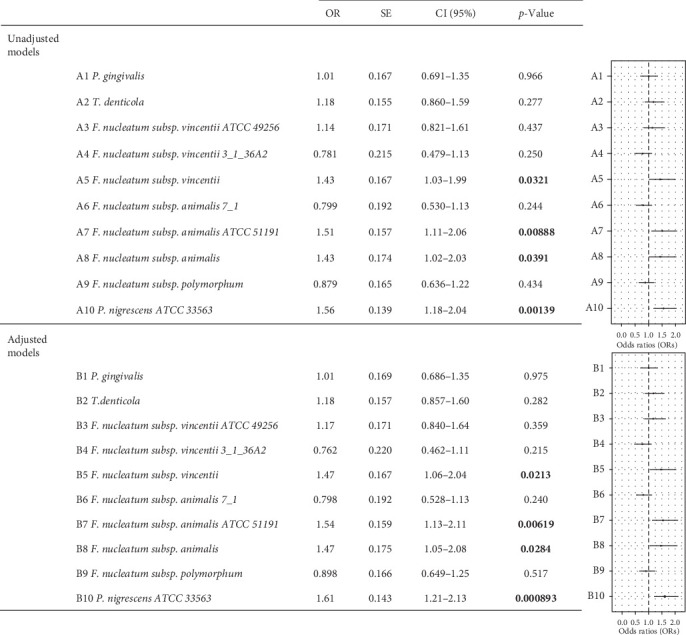
Logistic regressions result for taxa within the composite and elevated depressive symptoms. Significant positive associations between *P. nigrescens*, *F. nucleatum subsp. vincentii*, *F. nucleatum subsp. animalis ATCC 51191*, *F. nucleatum subsp. animalis*, and elevated depressive symptoms (*N* = 39), compared to minimal depressive symptoms (*N* = 397), both in unadjusted and adjusted models (all *p*  < 0.05). No evidence of associations with the other species. Adjusted models control for confounders: age, SES, diet, lifestyle risk, obesity, and hypertension. Error bars represent 95% CI. 95% CI, 95% confidence interval; OR, odds ratio estimate; SE, standard error; SES, socioconomic status. Significant *p*-values (*p* < 0.05) are in bold.

Several candidate oral pathogens within the composite were positively associated with elevated depressive symptoms (Figure 2). Species *P. nigrescens ATCC 33563* were significantly more abundant in participants with elevated depressive symptoms, compared to those with minimal depressive symptoms, both in the unadjusted (*p*  < 0.05) and adjusted (*p*  < 0.001) models, the latter controlling for age, SES, diet, lifestyle risk, obesity, and hypertension; an increase of one standard deviation of *P. nigrescens* (CLR-transformed) was associated with a 61% increase in the odds of having elevated depressive symptoms (1.61 [1.21, 2.13], *p*=0.000893, Figure 2; Supporting Information Figure S5). Three species of *F. nucleatum* were also positively associated with elevated depressive symptoms both in the unadjusted and adjusted models: *F. nucleatum subsp. animalis ATCC 51191* (1.54 [1.13, 2.11], *p*=0.00619, Figure 2; Supporting Information Figure S5), *F. nucleatum subsp. vincentii* (1.47 [1.06, 2.04], *p*=0.0213, Figure 2; Supporting Information Figure S5), and *F. nucleatum subsp. animalis* (1.47 [1.05, 2.08], *p*=0.0284, Figure 2; Supporting Information Figure S5). The abundances of *P. gingivalis*, *T. denticola*, and the other *F. nucleatum* species were not significantly associated with elevated depressive symptoms (all *p*  > 0.05, Figure 2). None of the candidate oral species was significantly associated with elevated anxiety symptoms, compared to minimal anxiety symptoms, in unadjusted or adjusted models (all *p*  > 0.05, Supporting Information Figure S6).

### 3.5. Association Between Oral Microbiota Diversity and Elevated Depressive and Anxiety Symptoms

**Figure 3:**
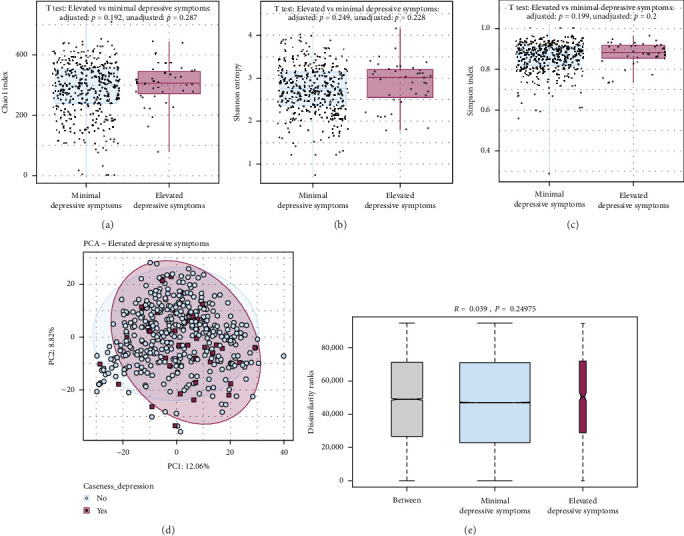
Alpha and beta diversities result in participants with elevated and minimal depressive symptoms. (a–c) Quantification of the variation in richness (Chao index) and diversity (Shannon entropy and Simpson index) of the oral microbiota between participants with minimal (*N* = 397) versus elevated (*N* = 39) depressive symptoms. No evidence of difference in richness/diversity (a-diversity) between the two groups, both in unadjusted and adjusted models (all *p*  > 0.05). The boxes represent the IQR; the error bars (whiskers) represent the full range of the data. (d, e) Principal component analysis (based on the Aitchison distance) between participants with minimal (*N* = 397) versus elevated (*N* = 39) depressive symptoms, and results of the related analysis of similarities (ANOSIM) comparing the oral microbiota between and within each group. No evidence of difference in dissimilarities (*β*-diversity) between the two groups (*p*  > 0.05). The boxes represent the IQR; error bars (whiskers) represent the full range of the data; box widths represent the number of samples in each group. IQR, interquartile range.

There were no differences in alpha diversity (Chao1 index, Shannon entropy, or Simpson index) between participants with elevated and minimal depressive symptoms (all *p*  > 0.05, Figure 3a–c), nor anxiety symptoms (all *p*  > 0.05, Supporting Information Figure S7), in unadjusted or adjusted models. Beta diversity showed no significant differences between participants with minimal and elevated depressive symptoms (ANOSIM test, *R* = 0.0392, *p*=0.210, Figure 3d,e), nor between participants with minimal and elevated anxiety symptoms (ANOSIM test, R = −0.0157, *p*=0.642, Supporting Information Figure S7).

### 3.6. Differential Abundance Analysis on All Oral Microbiota in the Samples

#### 3.6.1. Depressive Symptoms

When controlling for prespecified confounders (age, SES, diet, lifestyle risk, obesity, and hypertension), five oral species were positively associated with elevated depressive symptoms: *Streptococcus anginosus*, *Prevotella nigrescens ATCC 33563*, *Olsenella sp. oral taxon 807*, a *Campylobacter* uncultured bacterium, and a *Peptoniphilus* unidentified species (all OR > 1 with *q* < 0.1, Table 3). Notably, *S. anginosus* had an OR of 3.39 with *q* < 0.05 (Table 3; Supporting Information Figure S8), approximately equivalent to Cohen's *d* = 0.5 (i.e., a medium effect size) [49]. Moreover, five oral species were inversely associated with elevated depressive symptoms: *Streptococcus cristatus*, *Eikenella sp. NML130454*, *Capnocytophaga gingivalis ATCC 3362*, *Corynebacterium sp. oral clone AK153* and *Streptococcus respiraculi* (all OR < 1 with *q* < 0.1, Table 3). The unadjusted differential abundance analysis for depression produced no significant result (all *q* > 0.1), suggesting potential confounding by covariates, previously adjusted for in the multivariate analysis. Additionally, adjustment may have reduced variance due to covariate imbalance, improving statistical power to detect associations.

#### 3.6.2. Anxiety Symptoms

Two species were differentially abundant between participants with minimal and elevated anxiety symptoms when controlling for age, SES, diet, lifestyle risk, obesity, and hypertension: *Prevotella melaninogenica* and *Eikenella sp. NML130454* (*q* < 0.1, Table 4). These two oral species were inversely associated with elevated anxiety symptoms (both ORs < 1, Table 4). The unadjusted differential abundance analysis for anxiety produced no significant result (all *q* > 0.1).

## 4. Discussion

In this study of population-based men, we observed a modest, nonsignificant association between our oral pathogen composite and elevated depressive, but not anxiety, symptoms. We did not observe evidence of associations between oral microbiota alpha and beta diversity and depressive or anxiety symptoms. However, we did observe positive and inverse associations between various oral microbial taxa and both depressive symptoms and anxiety symptoms. Our analyses identified several oral species, *P. nigrescens*, *F. nucleatum subsp. animalis ATCC 51191*, *F. nucleatum subsp. animalis*, *F. nucleatum subsp. vincentii*, *S. anginosus*, *Olsenella sp. oral taxon 807*, a *Campylobacter* uncultured bacterium, and an unidentified species of an uncultured bacterium from the *Peptoniphilus* family, that were associated with elevated depressive symptoms. Conversely, several species showed an inverse association with depressive symptoms (*S. cristatus*, *Eikenella sp. NML130454*, *C. gingivalis ATCC 33624*, *Corynebacterium sp. oral clone AK15*3, and *S. respiraculi*), as well as anxiety symptoms (*P. melaninogenica* and *Eikenella sp. NML130454*).

There was a strong positive association between one of the oral species in our composite, *P. nigrescens* (*P. nigrescens ATCC 33563*) and elevated depressive symptoms. This finding is consistent with results from a recent study in patients with clinical depression, where *P. nigrescens* was more abundant in those with depression [15]. That study focused on young adults (18–38 years old), while our cohort comprised older participants (33–96 years old). Together, these results suggest that the association between *P. nigrescens* and elevated depressive symptoms may be relevant throughout adulthood. The pathogenicity of *P. nigrescens* includes its established link to periodontitis [50] and previously reported immune responses *in-vivo* [51]. This association warrants further investigation in larger cohorts and longitudinal studies to better understand its potential causality.

Three subspecies of *F. nucleatum* were positively associated with elevated depressive symptoms (*subsp. vincentii*, *subsp. animalis ATCC 51,191*, and *subsp. animalis*). Metagenomics enables the analysis of within-species differences, providing insights into potential differential effects of varying subspecies/strains [52]. Future research using metagenomic rather than 16S rRNA sequencing could further enhance our understanding of the complex relationships between *F. nucleatum* species and depressive symptoms and help determine whether these initial findings can be replicated.

Beyond the candidate oral pathogens, positive associations were found between several taxa and elevated depressive symptoms. These taxa were *S. anginosus*, *Olsenella sp. oral taxon 807*, a *Campylobacter* uncultured bacterium, and an unidentified species of an uncultured bacterium from the *Peptoniphilus* family. To our knowledge, these are novel associations not previously reported by similar studies [2, 15]. We note that *S. anginosus* bacteremia is associated with infections of the skin, soft tissue, and biliary tract [53]. These positive correlation findings align with and build on evidence that inflammatory markers may moderate the association between oral microbial composition and anxiety and depressive symptoms [2–6], potentially via indirect pathways such as LPS-induced neuroinflammation [51] and the vagus nerve [7], as well as through direct neural routes including the trigeminal nerve [9] and/or via the blood-brain barrier [10]. This supports the oral microbiota's emerging role in the microbiota–gut–brain axis and its relevance to common mental disorders [8].

Interestingly, several oral species in in our study were inversely associated with elevated depressive symptoms: *S. cristatus*, *Eikenella sp. NML130454*, *C. gingivalis ATCC 33624*, *Corynebacterium sp. oral clone AK15*3, and *S. respiraculi*. Elsewhere, inverse associations between many oral taxa and clinical depression or depressive symptoms have been reported [15]; however, none of the species previously identified overlapped with our results. These inverse associations may reflect potential protective roles or beneficial ecological interactions within the oral microbiome. For example, *S. cristatus* previously attenuated the *F. nucleatum*-induced interleukin-8 production in oral cells, suggesting a potential modulation of inflammatory responses to oral pathogens [54].

We report fewer associations between the oral microbiota and anxiety symptoms than observed for depressive symptoms. The species *P. melaninogenica* and *Eikenella sp. NML130454* showed an inverse association, where increased abundance was associated with lower anxiety symptoms. Depression and anxiety have different biological processes underpinning them. Inflammation is associated with an increased risk of clinical depression [6] and is thought to contribute to the development and persistence of depression through mechanisms involving cytokine signaling, neurotransmitter metabolism, and hypothalamic–pituitary–adrenal axis dysregulation [34]. Anxiety, on the other hand, is influenced by a more complex interplay of genetic, neurochemical, and structural factors [55], with inflammation playing a potential but less well-defined role to date.

We observed a modest, nonsignificant association between a composite of oral bacteria, comprising *P. gingivalis* [11–13], *F. nucleatum* [21], *T. denticola* [2, 9, 18–20], and *P. nigrescens* [15], and elevated depressive symptoms. The composite score analysis method accounts for the integrated effects of microbial functional guilds, rather than examining them in isolation [25, 28, 29]. This method is relatively recent, and none of the clinical studies on the oral microbiota in mental and neurodegenerative disorders discussed to date has employed this approach. Furthermore, there are no definitive guidelines for adapting a compositional data analysis approach to the development of a multiple-species composite score, and alternative methods for creating the composite could yield different results. Further refinement may be necessary to better understand which individual bacteria should be included in such a composite if this approach was to be pursued again.

We did not observe associations between either alpha or beta diversity and depressive or anxiety symptoms. This aligns with the findings in Australian young adults, where no disparities in alpha or beta diversity were reported between anxiety or depression groups [2]. However, other studies have presented contrasting results; for example, variations in alpha and beta diversity in the salivary microbiota were reported between individuals with higher psychological distress compared to controls [14], and differences in beta diversity were reported in the salivary microbiota of a clinically depressed cohort compared to controls [15].

This study represents a pioneering effort in understanding the association between oral microbiota and depressive and anxiety symptoms. The study methods adopted a causal modeling framework, controlling for potential confounding variables drawn from the literature. The study sample was not selected on the basis of a disease, and the large sample size (436 participants) provided robust control groups. The high quality of the oral microbiota data afforded information on 900 unique OTUs. Some identified to the subspecies level. However, the study has several limitations. First, there were few “cases” of elevated depressive symptoms (*n* = 39) in our sample, which limited our statistical power and may have accounted for the weak evidence arising from our primary analysis. Depression and anxiety disorders are nearly twice as prevalent in women [34, 55], and there may be disparities in oral microbiota between sexes [56]. Indeed, significant beta-diversity differences by sex were previously observed in adults with psychological distress [14]. The male-only sample also limits generalizability of our findings to women. The use of the HADS questionnaire, while consistent with previous literature, does not provide clinical diagnoses of depressive or anxiety disorders. Dichotomizing HADS scores for meaningful clinical representation may also have led to a general loss of statistical power in the study (increased type 2 error), while the cross-sectional, observational study design means that we cannot ascribe causality. We used a literature-informed additive composite to enhance interpretability of the cumulative pathogen burden and given our modest dataset size; with a larger dataset alternative approaches such as PCA could identify latent patterns or underlying structures in the data. Differences in oral bacteria across niches and study-specific swabbing protocols could introduce variability [57]. This limitation is not unique to this study; methodological heterogeneity is a feature of the oral and broader microbiota field at large [58, 59]. Regarding data collection, factors like mouthwash use, food or beverage consumption, and tooth brushing habits were not controlled, potentially influencing the oral microbiota composition [60, 61]. Finally, the study's relatively narrow age range, with predominantly middle-aged and older individuals, may not reflect younger populations [35, 38], and the regional nature of the cohort may also limit generalizability [62].

## 5. Conclusion

This study provides new evidence showing associations between oral microbiota species and symptoms of common mental disorders, particularly for depressive symptoms. While several species—such as *P. nigrescens—*were positively associated with elevated depressive symptoms, others, like *S. cristatus*, had inverse associations with both elevated depressive and anxiety symptoms. Further research replicating our findings and seeking clarity on the mechanistic pathways involved is needed, ideally in larger sample sizes of both sexes.

## Figures and Tables

**Table 1:** Description of the covariates that were adjusted for in the current study.

Covariates	Data source	Handling/computation
Age	Participants self-reported date of birth	Age on assessment day
Socioeconomic status (SES)	Socioconomic Indexes for Areas (Australian Bureau of Statistics)	Deciles (1–10) of the IRSAD
Diet	DQES questionnaire (Cancer Council Victoria)	E-DII score
Lifestyle risk (smoking, alcohol intake, and exercise)	Questionnaire on smoking, current alcohol intake, and physical activity	Lifestyle risk score (0–5)
Obesity	Participants self-reported height and weight	Body mass index (BMI) ≥30
Hypertension	Participants self-reported hypertension	/

*Note:* The computation of the lifestyle risk score is detailed in Supporting Information Table S1; missing covariate data were imputed to the median of the cohort.

Abbreviations: DQES, Dietary Questionnaire for Epidemiological Studies; E-DII, Energy-adjusted Dietary Inflammatory Index; IRSAD, Index of Relative Socioeconomic Advantage and Disadvantage.

**Table 2:** Participant characteristics.

	All participants (*N* = 436)		Minimal depressive symptoms (*N* = 397)	Elevated depressive symptoms (*N* = 39)	Minimal anxiety symptoms (*N* = 370)	Elevated anxiety symptoms (*N* = 66)
Age (y)	62 (50, 71)		62 (51, 71)	57 (50, 74)	63 (52, 72)	54 (44, 68)
SES: IRSAD deciles	6 (4, 7)		6 (4, 8)	5 (3, 7)	6 (4, 7)	6 (3, 7)
Diet: E-DII	−0.07 (−0.90, 0.63)		−0.07 (−0.90, 0.62)	−0.23 (−0.91, 0.68)	−0.11 (−0.98, 0.58)	0.04 (−0.45, 0.81)
Lifestyle risk: smoking, alcohol and exercise	2 (2, 3)		2 (2, 3)	3 (2, 3)	2 (2, 3)	3 (2, 3)
Chronic disorder: Obesity	114 (26.15%)		103 (25.9%)	11 (28.2%)	96 (25.9%)	18 (27.3%)
Chronic disorder: Hypertension	147 (33.72%)		131 (33.0%)	16 (41.0%)	126 (34.1%)	21 (31.8%)

Pathogen composite (CLR)	9 (2, 17)		9 (2, 16)	11 (6, 18)	9 (2, 17)	10 (4, 17)

Elevated depressive symptoms: HADS-D ≥ 8	39 (8.94%)		—	—	20 (5.4%)	19 (28.8%)
Elevated anxiety symptoms: HADS-A ≥ 8	66 (15.14%)		47 (11.8%)	19 (48.7%)	—	—

*Note:* Description of all eligible participants (*N* = 436), including those with minimal and elevated depressive symptoms, as well as those with minimal and elevated anxiety symptoms. The pathogen composite reflects the combined load of *P. gingivalis*, *T. denticola*, *F. nucleatum subsp. vincentii ATCC 49256*, *F. nucleatum subsp. vincentii 3_1_36A2*, *F. nucleatum subsp. vincentii*, *F. nucleatum subsp. animalis 7_1*, *F. nucleatum subsp. polymorphum*, *F. nucleatum subsp. animalis ATCC 51191*, *F. nucleatum subsp. animalis* and *P. nigrescens ATCC 33563*. Median (IQR); n (%).

Abbreviations: CLR, centered log ratio; E-DII, energy-adjusted dietary Inflammatory Index; HADS-D/A, Depression/Anxiety score from the Hospital Anxiety and Depression Scale questionnaire; IQR, interquartile range; IRSAD, Index of Relative Socioeconomic Advantage and Disadvantage; SES, socioeconomic status.

**Table 3:** Significant results (*q* < 0.1) of the differential abundance analysis across all oral taxa at the species level for depressive symptoms, controlling for confounders.

Phylum–Class–Order–Family–Genus	Species	OR	SE	*q*-Value	*p*-Value
*Firmicutes–Bacilli–Lactobacillales–Streptococcaceae-Streptococcus*	*Streptococcus anginosus*	3.39	0.380	0.0450	0.00139
*Bacteroidota–Bacteroidia–Bacteroidales–Prevotellaceae–Prevotella*	*Prevotella nigrescens ATCC 33563*	2.04	0.225	0.0501	0.00166
*Actinobacteriota–Coriobacteriia–Coriobacteriales–Atopobiaceae–Olsenella*	*Olsenella sp. oral taxon 807*	2.44	0.291	0.0606	0.00233
*Firmicutes–Bacilli–Lactobacillales–Streptococcaceae–Streptococcus*	*Streptococcus cristatus*	0.337	0.364	0.0713	0.00297
*Proteobacteria–Gammaproteo–bacteria–Burkholderiales–Neisseriaceae–Eikenella*	*Eikenella sp. NML130454*	0.525	0.217	0.0756	0.00321
*Bacteroidota–Bacteroidia–Flavobacteriales–Flavobacteriaceae–Capnocytophaga*	*Capnocytophaga gingivalis ATCC 33624*	0.506	0.231	0.0781	0.00335
*Campilobacterota–Campylobacteria–Campylobacterales–Campylobacteraceae–Campylobacter*	*uncultured bacterium*	2.05	0.251	0.0906	0.00440
*Firmicutes–Clostridia–Peptostreptococcales–Tissierellales–Peptoniphilus–uncultured bacterium*	*unidentified*	1.50	0.143	0.0954	0.00484
*Actinobacteriota–Actinobacteria–Corynebacteriales–Corynebacteriaceae–Corynebacterium*	*Corynebacterium sp. oral clone AK153*	0.521	0.231	0.0990	0.00507
*Firmicutes–Bacilli–Lactobacillales–Streptococcaceae–Streptococcus*	*Streptococcus respiraculi*	0.653	0.151	0.0993	0.00512

*Note:* Species are ordered based on ascending *q*-values. All tests are adjusted for confounders: age, SES, diet, lifestyle risk, obesity, and hypertension.

OR, odds ratio estimate; SE, standard error; *q*-value: the *p*-value after Benjamini–Hochberg adjustment for multiple hypothesis testing; SES, Socioeconomic status.

**Table 4:** Significant results (*q* < 0.1) of the differential abundance analysis across all oral taxa at the species level for anxiety symptoms, controlling for confounders.

*Phylum–Class–Order–Family–Genus*	*Species*	OR	SE	*q*-Value	*p*-Value
*Bacteroidota–Bacteroidia–Bacteroidales–Prevotellaceae–Prevotella*	*Prevotella melaninogenica*	0.641	0.145	0.0657	0.00242
*Proteobacteria–Gammaproteobacteria–Burkholderiales–Neisseriaceae–Eikenella*	*Eikenella sp. NML130454*	0.602	0.175	0.0872	0.00399

*Note:* Species are ordered based on ascending *q*-values. Both tests are adjusted for confounders: age, SES, diet, lifestyle risk, obesity, and hypertension.

OR, odds ratio estimate; SE, standard error; *q*-value, the *p*-value after Benjamini–Hochberg adjustment for multiple hypothesis testing; SES, socioeconomic status.

## Data Availability

The data that support the findings of this study are available upon request from the corresponding author. The data are not publicly available due to privacy or ethical restrictions.

## Nomenclature

HADS: Hospital Anxiety and Depression Scale
CLR: Centered log ratio
OTUs: Operational taxonomic units
SES: Socioeconomic status
IRSAD: Index of Relative Socioeconomic Advantage and Disadvantage
E-DII: Energy-Adjusted Dietary Inflammatory Index
OR: Odds ratio
CI: Confidence interval.

## Supporting Information

Supporting Information The file provides additional details on the study covariates, participant inclusion/exclusion, oral species prevalence in the samples, a correlation plot of oral species, abundance of the oral pathogen composite in the samples, as well as several supplementary results. This includes Tables S1–S3 and Figures S1–S8.
