# Supplementary material for: Associations Between Oral Microbiota Pathogens and Elevated Depressive and Anxiety Symptoms in Men
Source: Depress Anxiety. 2025 Jul 21;2025:9961595. doi: 10.1155/da/9961595 (PMC12303655; doi:10.1155/da/9961595)
Supplement: Supporting Information — The file provides additional details on the study covariates, participant inclusion/exclusion, oral species prevalence in the samples, a correlation plot of oral species, abundance of the oral pathogen composite in the samples, as well as several supplementary results. This includes Tables S1–S3 and Figures S1–S8. [file 9961595.f1.pdf]

Supplementary material

Associations between oral microbiota pathogens and elevated depressive and anxiety symptoms in men

Fannie Kerff, Julie A Pasco, Lana J Williams, Felice N Jacka, Amy Loughman, Samantha L Dawson

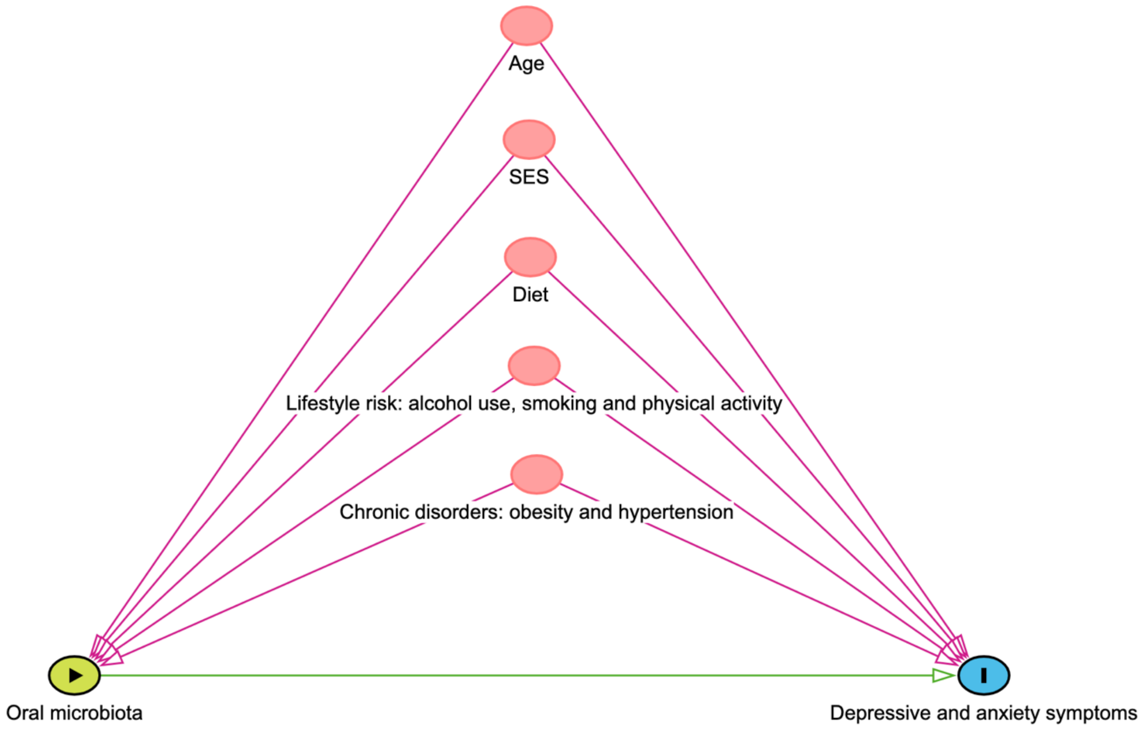

Supplementary Fig. S1. **Directed Acyclic Graph**.
A priori confounding variables in this analysis: age, SES, diet, lifestyle risk (alcohol use, smoking and physical activity) and the presence of chronic disorders (especially, obesity and hypertension). SES, Socio-Economic Status.

Component variable	No risk (0)	Low risk (1)	High risk (2)
Smoking	Never	Former	Current
Current alcohol intake	Abstinence	Modest	Heavy
Physical activity	Active	Inactive	

Notes. Definition of the LRS component variables: smoking, current alcohol intake and physical activity.

Supplementary Table S1. **Measure of the lifestyle risk score**.
The score ranges from 0 to 5; a score of 5 corresponds to the maximum risk. This combines information on smoking status, current alcohol intake and physical activity in the participants. Smoking status in the individuals was defined as never (0; no risk), former (1; low risk), or current (2; high risk). Current alcohol intake included three groups: abstinent i.e., no drinks/week (0; no risk), modest i.e., 1–7 drinks/week (1; low risk), and heavy i.e., > 7 drinks/week (2; high risk) drinkers. A drink was defined as 14 grams of alcohol (National Institute of Alcohol Abuse and Alcoholism). Finally, physical activity was defined as active (0; no risk) or inactive (1; low risk). Physical activity was computed using a combination of the Baecke (< 60 years-old (Baecke et al., 1982)) and Voorrips (>60 years-old (elderly) (Voorrips et al., 1991)) questionnaires dichotomized at the median.

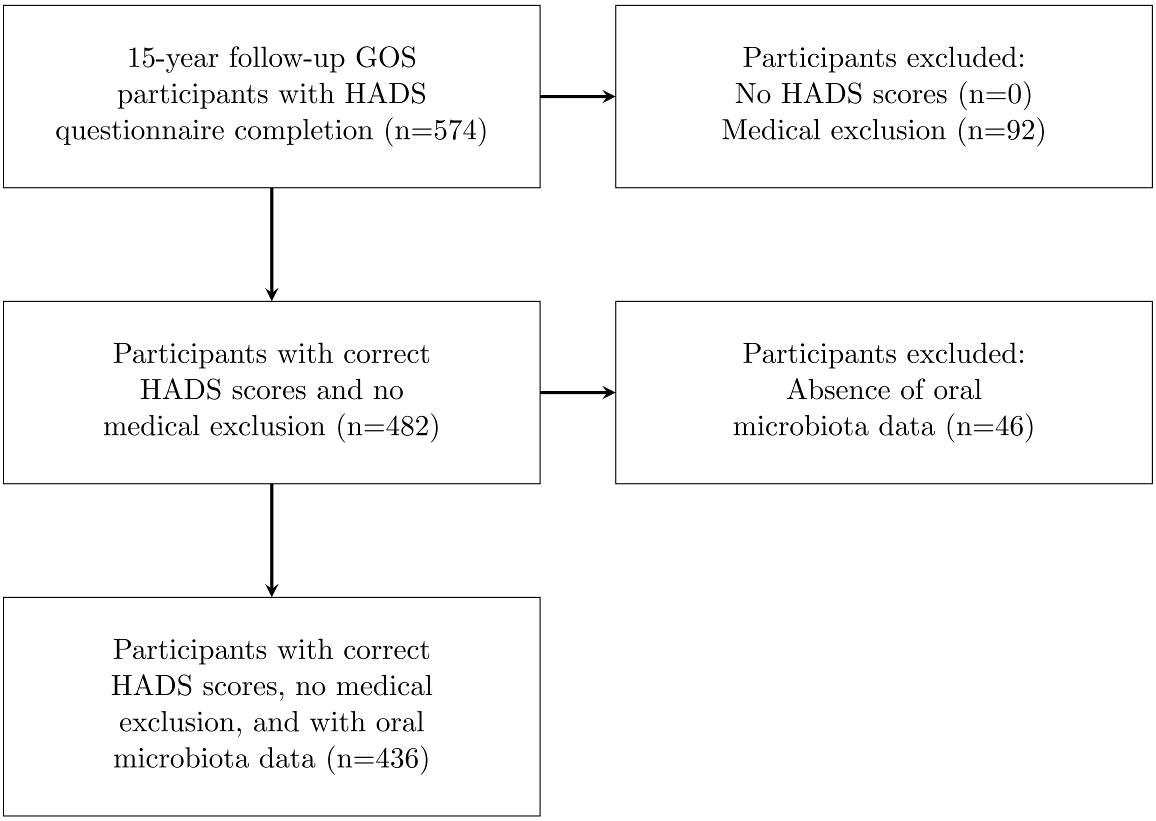

Supplementary Fig. S2. **Flow chart of the inclusion and exclusion process of the participants**. Description of the selection of the 436 participants included in the analysis. GOS, Geelong Osteoporosis Study; HADS, Hospital Anxiety and Depression Scale.

	Past-year conditions					Long-term conditions		*Total (omitting duplicates)*
	Cancer	Severe heart disease	Severe brain disease	Severe liver disease	Severe kidney disease	Brain conditions	Heart conditions	
	Lung, bowel, breast, uterus, cervix, throat, brain, melanoma, non-melanoma skin, leukemia, myeloma, lymphoma, prostate, bone marrow lymphoma, clear cell carcinoma of the kidney, pituitary gland tumor	Heart attack (myocardial infarction), stroke, heart disease, heart bypass surgery, heart pacemaker insertion, stents in heart, leaky or defects in valve and aorta, valve replacement, myocardial bridging and ectopic heartbeat, hole in the heart	Brain damage, brain injury, brain aneurysm, and subarachnoid hemorrhage	Liver hepatic failure and cirrhosis liver	Kidney renal failure	Parkinson's disease, Alzheimer's disease	Hole in heart since birth	
Total	53	31	4	2	4	3	2	92 (16%)

Supplementary Table S2. **Severe medical conditions excluded from the analysis**.
List of the medical conditions that led to the exclusion of the participants. 92 participants (16%) were excluded in total.

Species	Prevalence (# of samples)	Prevalence (% of samples)	Correlation with the composite	Included in pathogen composite?
Fusobacterium nucleatum subsp. polymorphum	390	89.4 %	0.5	Yes
Fusobacterium nucleatum subsp. vincentii ATCC 49256	372	85.3 %	0.8	Yes
Fusobacterium nucleatum subsp. animalis	364	83.5 %	0.8	Yes
Fusobacterium nucleatum subsp. vincentii	327	75.0 %	0.7	Yes
Fusobacterium nucleatum subsp. animalis ATCC 51191	287	65.8 %	0.7	Yes
Treponema denticola	193	44.3 %	0.5	Yes
Fusobacterium nucleatum subsp. animalis 7_1	177	40.6 %	0.5	Yes
Prevotella nigrescens ATCC 33563	171	39.2 %	0.4	Yes
Fusobacterium nucleatum subsp. vincentii 3_1_36A2	71	16.3 %	0.2	Yes
Fusobacterium nucleatum subsp. fusiforme ATCC 51190	51	11.7 %	*0*	*No*
Porphyromonas gingivalis	47	10.8 %	0.1	Yes
Aggregatibacter actinomycetemcomitans D7S-1	10	*2.29 %*	*-0.2*	*No*
Aggregatibacter actinomycetemcomitans DSM 8324	10	*2.29 %*	*-0.3*	*No*
Aggregatibacter actinomycetemcomitans	2	*0.46 %*	*-0.4*	*No*
Klebsiella pneumoniae	*0*	*0 %*	*/*	*No*

Supplementary Table S3. **Candidate oral species for the pathogen composite: prevalence in the samples and correlation with the composite.**
Prevalence reported both as raw number of samples and as percentage of samples the species are detected in. Correlations between each species and the pathogen composite are reported.

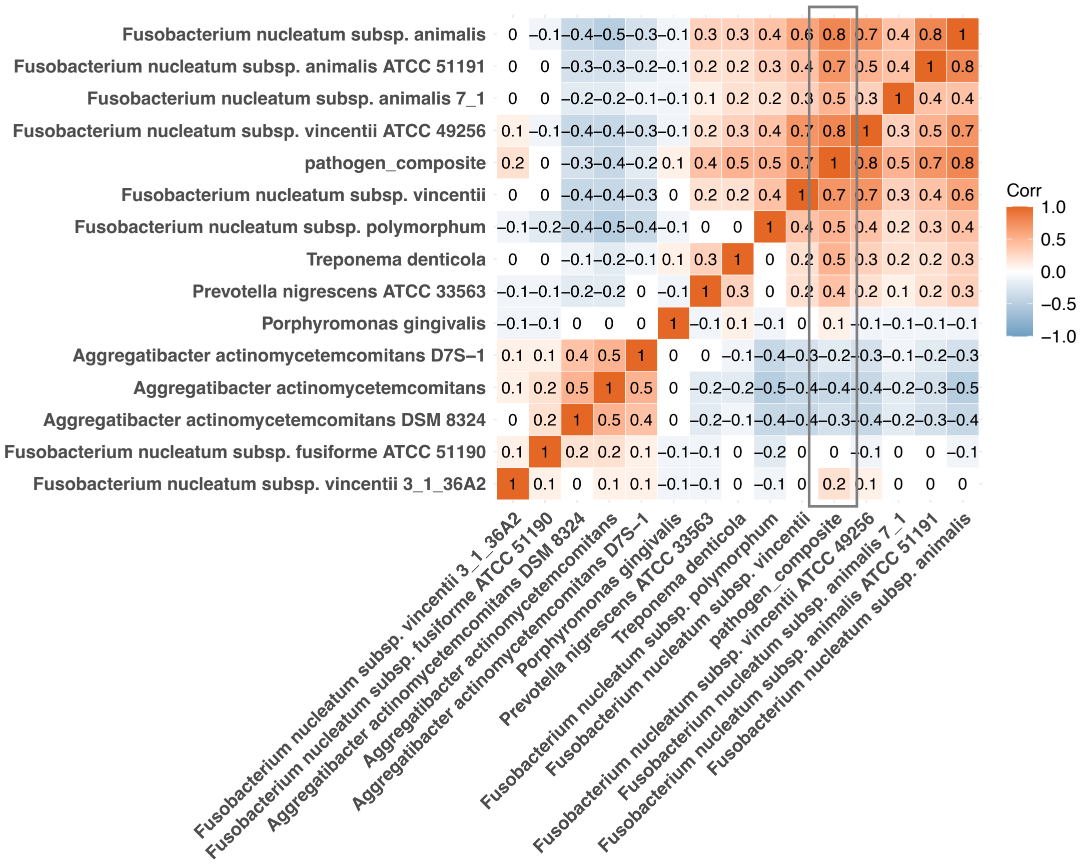

Supplementary Fig. S3. **Correlation matrix of the species within the pathogen composite.** The numbers represent the correlation coefficients of species CLR-transformed abundances. All three *Aggregatibacter actinomycetemcomitans* species and *Fusobacterium nucleatum subsp. fusiforme ATCC 51190* species are not positively correlated with the pathogen composite (correlation ≤ 0). CLR, Centered Log Ratio.

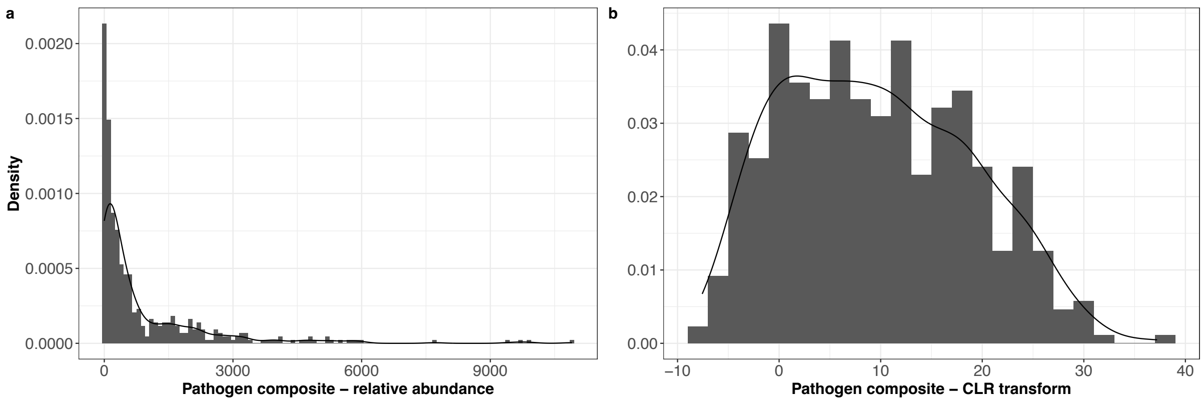

Supplementary Fig. S4. **Histograms and density curves of the distributions of the oral pathogen composite.**

a Relative abundance. **b** CLR transformed abundance. The pathogen composite comprises species *P. gingivalis*, *F. nucleatum* (excluding *F. nucleatum subsp. fusiforme ATCC 51190*), *T. denticola*, and *P. nigrescens*. CLR, Centered Log Ratio.

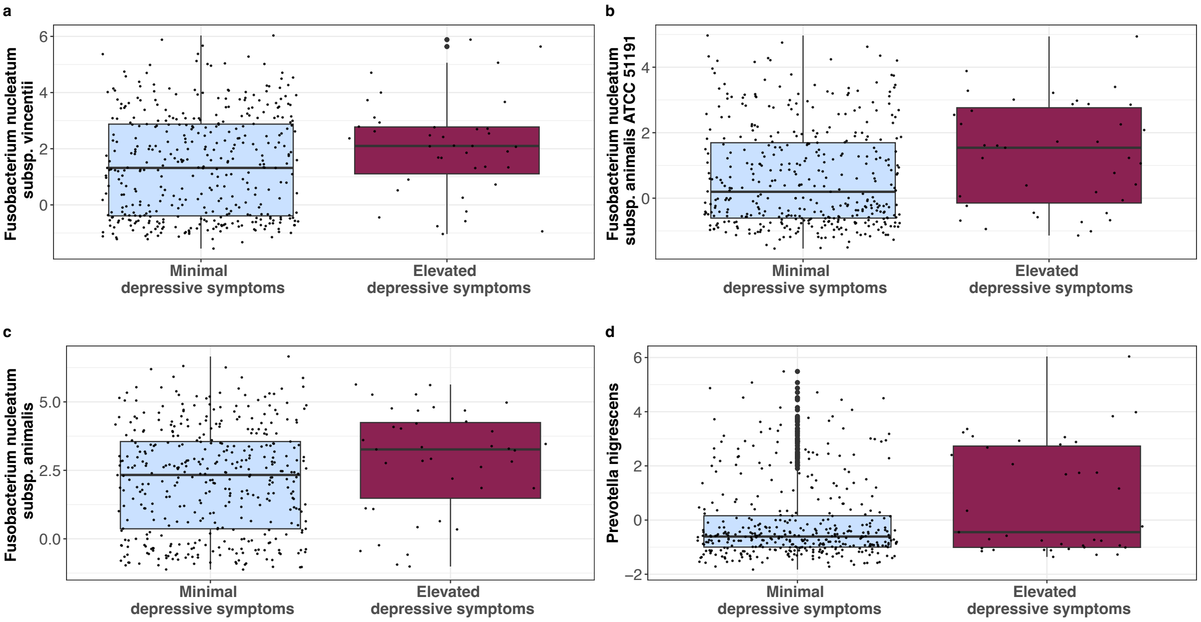

Supplementary Fig. S5. **Distributions of species in participants with minimal and elevated depressive symptoms (CLR transformed abundance).**
a *F. nucleatum subsp. vincentii*. **b** *F. nucleatum subsp. animalis ATCC 51191*. **c** *F. nucleatum subsp. animalis*. **d** *P. nigrescens.* The different distributions in the sub-samples illustrates the significantly greater abundances of *F. nucleatum subsp. vincentii*, *F. nucleatum subsp. animalis ATCC 51191*, *F. nucleatum subsp. animalis*, and *P. nigrescens* species in participants with elevated depressive symptoms. The boxes represent the IQR; the error bars (whiskers) represent the range of data within 1.5 times the IQR above the third quartile and below the first quartile. CLR, Centered Log Ratio; IQR, interquartile range.

			OR	SE	CI (95%)	p-value
Unadjusted models						
	A1	*P. gingivalis*	0.821	0.167	0.564 - 1.10	0.237
	A2	*T. denticola*	1.16	0.127	0.894 - 1.47	0.255
	A3	*F. nucleatum subsp. vincentii ATCC 49256*	1.08	0.135	0.830 - 1.41	0.575
	A4	*F. nucleatum subsp. vincentii 3_1_36A2*	0.799	0.163	0.560 - 1.07	0.170
	A5	*F. nucleatum subsp. vincentii*	1.21	0.133	0.932 - 1.57	0.152
	A6	*F. nucleatum subsp. animalis 7_1*	0.885	0.142	0.659 - 1.16	0.392
	A7	*F. nucleatum subsp. animalis ATCC 51191*	1.27	0.128	0.983 - 1.63	0.0645
	A8	*F. nucleatum subsp. animalis*	1.21	0.135	0.930 - 1.58	0.157
	A9	*F. nucleatum subsp. polymorphum*	1.06	0.135	0.817 - 1.39	0.657
	A10	*P. nigrescens ATCC 33563*	1.19	0.124	0.922 - 1.50	0.168
Adjusted models						
	B1	*P. gingivalis*	0.813	0.166	0.559 - 1.09	0.215
	B2	*T. denticola*	1.14	0.130	0.880 - 1.47	0.298
	B3	*F. nucleatum subsp. vincentii ATCC 49256*	1.05	0.141	0.798 - 1.39	0.730
	B4	*F. nucleatum subsp. vincentii 3_1_36A2*	0.816	0.167	0.567 - 1.10	0.225
	B5	*F. nucleatum subsp. vincentii*	1.19	0.137	0.909 - 1.56	0.204
	B6	*F. nucleatum subsp. animalis 7_1*	0.867	0.145	0.642 - 1.14	0.325
	B7	*F. nucleatum subsp. animalis ATCC 51191*	1.28	0.134	0.981 - 1.66	0.0665
	B8	*F. nucleatum subsp. animalis*	1.18	0.141	1.894 - 1.56	0.246
	B9	*F. nucleatum subsp. polymorphum*	1.06	0.140	0.809 - 1.40	0.667
	B10	*P. nigrescens ATCC 33563*	1.19	0.129	0.914 - 1.52	0.186

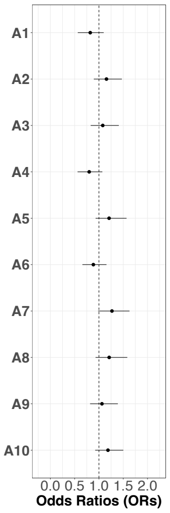

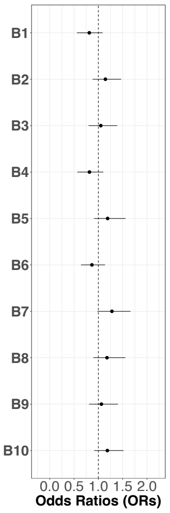

Supplementary Fig. S6. **Logistic regressions result for species within the composite and elevated anxiety symptoms.**
No significant association between any species and elevated anxiety symptoms (N=66), compared to minimal depressive symptoms (N=370), both in unadjusted and adjusted models (all $p > 0.05$). Adjusted models control for confounders: age, SES, diet, lifestyle risk, obesity and hypertension. Error bars represent CI (95%). OR, Odds Ratio estimate; SE: Standard Error; CI (95%): 95% Confidence Interval; SES, Socio-Economic Status.

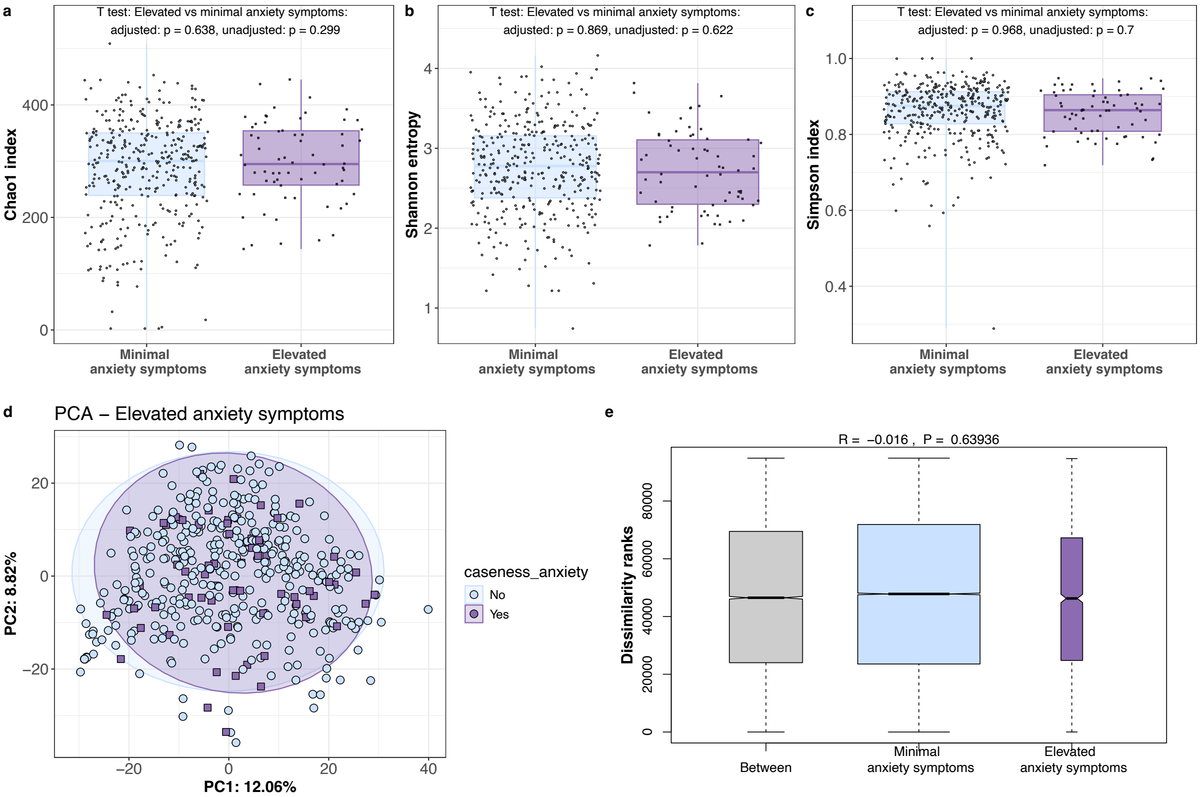

Supplementary Fig. S7. **Alpha and beta diversities result in participants with elevated and minimal anxiety symptoms.**

(**a-c**) Quantification of the variation in richness (Chao index) and diversity (Shannon entropy and Simpson index) of the oral microbiota between participants with minimal (N=370) versus elevated (N=66) anxiety symptoms. No evidence of difference in richness/diversity (α-diversity) between the two groups, both in unadjusted and adjusted models (all $p > 0.05$). The boxes represent the IQR; the error bars (whiskers) represent the full range of the data. (**d-e**) Principal component analysis (based on the Aitchison distance) between participants with minimal (N=370) versus elevated (N=66) anxiety symptoms, and results of the related analysis of similarities (ANOSIM) comparing the oral microbiota between and within each group. No evidence of difference in dissimilarities (β-diversity) between the two groups ($p > 0.05$). The boxes represent the IQR; error bars (whiskers) represent the full range of the data; box widths represent the number of samples in each group. IQR, interquartile range.

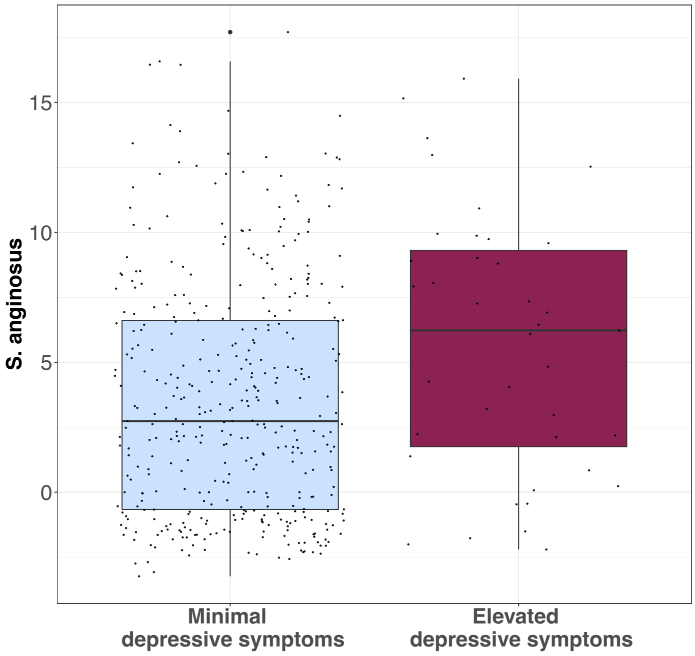

Supplementary Fig. S8. **Distributions of *Streptococcus anginosus* in participants with minimal and elevated depressive symptoms (CLR transformed abundance).**
The large difference in the medians of both samples illustrates this significantly greater abundance of *S. anginosus* in the participants with elevated depressive symptoms. The boxes represent the IQR; the error bars (whiskers) represent the range of data within 1.5 times the IQR above the third quartile and below the first quartile. CLR, Centered Log Ratio; IQR, interquartile range.

Supplementary references

Baecke, J., Burema, J., Frijters, J., 1982. A short questionnaire for the measurement of habitual physical activity in epidemiological studies. The American Journal of Clinical Nutrition 36, 936–942. https://doi.org/10.1093/ajcn/36.5.936

Voorrips, L.E., Ravelli, A.C., Dongelmans, P.C., Deurenberg, P., Van Staveren, W.A., 1991. A physical activity questionnaire for the elderly. Med Sci Sports Exerc 23, 974–979.
